# The Effect of Neutrophil-to-Lymphocyte Ratio on Prognosis in Malignant Ovarian Germ Cell Tumors

**DOI:** 10.3390/diagnostics15081040

**Published:** 2025-04-19

**Authors:** Yagmur Arslan, Ipek Betul Ozcivit Erkan, Atacem Mert Aytekin, Cansu Turker Saricoban, Abdullah Serdar Acikgoz, Tugan Bese, Oguzhan Kuru

**Affiliations:** 1 Department of Obstetrics and Gynecology, Cerrahpasa Faculty of Medicine, Istanbul University-Cerrahpasa, Istanbul 34098, Türkiye; ipekbetul.ozcivit@ogr.iuc.edu.tr; 2 Division of Gynecologic Oncology, Department of Obstetrics and Gynecology, Cerrahpasa Faculty of Medicine, Istanbul University-Cerrahpasa, Istanbul 34098, Türkiye; atacem.aytekin@iuc.edu.tr (A.M.A.); abdullahserdar.acikgoz@iuc.edu.tr (A.S.A.); tuganbese@gmail.com (T.B.); oguzhan.kuru@iuc.edu.tr (O.K.); 3 Department of Pathology, Cerrahpasa Faculty of Medicine, Istanbul University-Cerrahpasa, Istanbul 34098, Türkiye; cansu.saricoban@iuc.edu.tr

**Keywords:** malignant ovarian germ cell tumors, ovarian cancer, overall survival, disease-free survival, prognostic marker, neutrophil-to-lymphocyte ratio (NLR), ROC curve, inflammatory biomarkers, preoperative NLR

## Abstract

**Background/Objectives**: Ovarian germ cell tumors are rare, and determining prognostic factors is crucial for individualizing management strategies. We aimed to determine an optimal neutrophil-to-lymphocyte ratio (NLR) cut-off value for predicting survival outcomes in malignant ovarian germ cell tumors, and to evaluate the prognostic significance of NLR in these tumors. **Methods**: This retrospective cohort study included women diagnosed with malignant ovarian germ cell tumors who underwent surgery at Istanbul University-Cerrahpasa between 2000 and 2024. Patients with benign tumors; incomplete follow-up; inaccessible data; history of hematological or rheumatic diseases; inflammatory conditions such as diabetes mellitus, asthma, or renal failure; as well as those with acute/chronic infections or sepsis were excluded. Data collected included demographic characteristics, surgical and pathological findings, chemotherapy details, disease progression, survival outcomes, and laboratory values at preoperative, postoperative, and post-chemotherapy time points. The NLR was calculated and compared for overall survival and disease-free survival. **Results**: The study included 44 patients with a pathologically confirmed diagnosis of malignant ovarian germ cell tumors. The NLR cut-off value for survival prediction was determined as 3.69 using the ROC curve. The effect of preoperative NLR on overall survival was found to be significant. The median overall survival was significantly lower in the group with NLR ≥ 3.69 (153.2 months) compared to the group with NLR < 3.69 (234 months) (*p* = 0.010). However, there was no statistically significant difference in median disease-free survival between the NLR ≥ 3.69 group (159.3 months) and the NLR < 3.69 group (215 months). **Conclusions**: The preoperative NLR was found to have a significant impact on overall survival but not on disease-free survival. A cut-off value of 3.69 can be used to assess short survival time.

## 1. Introduction

Ovarian germ cell tumors are rare tumors originating from primordial germ cells, accounting for approximately 20–25% of all ovarian tumors, with an incidence of 2.34–3.4/1,000,000 [1,2,3]. Female ovarian germ cell tumors are commonly seen in young women between the ages of 10 and 30, with 5% being malignant. Dysgerminoma is the most common malignant type [3]. The main presenting symptom is an adnexal mass with rapid growth in prepubertal and adolescent patients, which may be accompanied by necrosis, intra-abdominal hemorrhage, rupture, or torsion, leading to acute abdomen symptoms [4]. However, they may also remain asymptomatic [5]. The diagnostic process should begin with a pelvic ultrasound, followed by tumor marker assessment, including alpha-fetoprotein (AFP), beta-human chorionic gonadotropin (hCG), lactate dehydrogenase (LDH), and, if indicated, serum cancer antigen 125 (CA125). Additional imaging of the abdomen, pelvis, and chest may be performed when clinically indicated [4]. Management and follow-up should be conducted in accordance with current guidelines [4].

Identifying prognostic factors is essential for risk-adapted treatment planning and individualizing both initial and relapse management strategies in malignant ovarian germ cell tumors. Commonly used tumor markers such as beta-hCG and AFP, along with the initial International Federation of Gynecology and Obstetrics (FIGO) stage, can help assess treatment response, guide follow-up, and predict overall survival (OS) [6]. However, simpler and more cost-effective prognostic markers derived from routine preoperative blood tests are more practical to use in clinical practice [7,8].

Tumors induce systemic inflammation by activating neutrophils, which secrete cytokines such as tumor necrosis factor-alpha (TNF-alpha), interleukin-2 (IL-2), IL-6, and IL-10. These cytokines contribute to the formation of a tumor-supportive microenvironment by promoting cancer progression and suppressing the lymphocytic immune response. Neutrophils contain and release vascular endothelial growth factor (VEGF), a key pro-angiogenic factor that plays a crucial role in tumor development [9]. S100A8 and S100A9 are calcium-binding immunogenic proteins primarily secreted by neutrophils and known for their role as pro-inflammatory mediators. Recent studies have shown that these proteins are overexpressed in tumor cells and play a key role in tumor survival by promoting development, growth, and metastasis through disruption of tumor metabolism and suppression of immune cell tumoricidal activity [10,11,12]. Given the role of systemic inflammation in cancer, inflammation-related markers have been investigated, as inflammation contributes to ovarian cancer cell seeding, metastasis, the reactivation of dormant cancer cells, and early recurrence [13,14,15,16]. Through these mechanisms, neutrophils are closely linked to ovarian tumor progression and metastasis [7]. In mouse models, ovarian cancer cells exhibit increased expression of neutrophil-related chemokines and neutrophil expansion [17,18]. Notably, S100A8 has also been identified as a potential non-invasive biomarker, with previous studies proposing its use as a salivary protein for the early diagnosis of ovarian cancer [19].

Neutrophil-to-lymphocyte ratio (NLR) has been investigated as a simple inflammatory marker in ovarian cancer. NLR has been shown to be associated with OS and disease-free survival (DFS) in various cancer types, including colorectal, gastric, liver, pancreatic, breast, urological, ovarian, endometrial, and cervical cancers [20,21,22,23,24,25,26]. High preoperative NLR in peripheral blood is associated with decreased OS in epithelial ovarian cancer (EOC) and correlates with advanced tumor grade, stage, bilateral tumors, and poor prognosis [7,23,27,28]. Elevated NLR reflects neutrophil-driven immune suppression, leading to relative lymphocytopenia and increased recurrence risk [7,29]. While NLR has been associated with survival outcomes in various cancers, its prognostic significance in female malignant ovarian germ cell tumors remains unclear. This study aims to evaluate the prognostic value of NLR in female malignant ovarian germ cell tumors and to establish an optimal NLR cut-off value for predicting survival outcomes.

## 2. Materials and Methods

### 2.1. Study Design and Study Population Selection

In this retrospective cohort study, we analyzed women who underwent surgery at the Subdivision of Gynecological Oncology, Department of Obstetrics and Gynecology, Istanbul University-Cerrahpasa, Cerrahpasa Faculty of Medicine, between 2000 and 2024, with a pathological diagnosis of malignant ovarian germ cell tumors. Exclusion criteria included women whose diagnosis, surgery, or follow-up was not conducted in our department; those with benign ovarian germ cell tumors, such as mature teratomas; patients who discontinued follow-up for any reason or had inaccessible data; and those with a history of hematological or rheumatic diseases, inflammatory conditions such as diabetes mellitus, asthma, or renal failure, as well as those with acute/chronic infections or sepsis that could affect NLR values.

Detailed patient histories were recorded from the electronic database, including age; body mass index (BMI); presence of pregnancy; comorbidities; type and date of surgery; initial tumor size; chemotherapy regimen, duration and number of chemotherapy cycles; lymph node involvement; postoperative residual tumor and its size (cm); histopathological findings (presence of dysgerminoma, pathological subtype, lymphovascular space invasion (LVSI), ovarian capsule invasion, presence of necrosis); presence of ascites; cause of death; total follow-up duration; disease-free survival duration; recurrence status (including time and site, if present); FIGO stage [30]; metastasis status (including site, if present); and chemotherapy side effects (including the type of side effect, if present). Patient mortality status and date of death were obtained from the death notification system [31]. DFS is defined as the duration after successful treatment during which no symptoms or effects of the disease appear [32]. OS is defined as the time from the date of diagnosis until the last follow-up or the date of death [33].

Preoperative, postoperative, and post-chemotherapeutic laboratory values were retrieved from the electronic database, including hemoglobin (Hb); hematocrit (Htc); platelet (Plt); neutrophil; lymphocyte and white blood cell (WBC) count; red cell distribution width (RDW); lactate dehydrogenase (LDH); beta-hCG; carcinoembryonic antigen (CEA); AFP; cancer antigen-125 (CA-125); CA 19-9; and CA 15-3.

The neutrophil and lymphocyte counts were obtained from a complete blood count analysis of venous blood samples collected in EDTA tubes and analyzed using the Sysmex XN-2000 (Sysmex Corporation, Kobe, Japan). The NLR was calculated by dividing the neutrophil count by the lymphocyte count. Preoperative, postoperative, and post-chemotherapy values were measured within one month before surgery, one month after surgery, and one month after chemotherapy, respectively.

### 2.2. Outcomes

The primary outcomes of the study were DFS and OS in relation to preoperative, postoperative, and post-chemotherapy NLR values. Secondary outcomes included other factors influencing DFS and OS, such as age, FIGO stage, comorbidities, postoperative residual disease, chemotherapy response, lymph node involvement, and additional laboratory markers analyzed for their association with survival and recurrence.

### 2.3. Statistical Analysis

Descriptive statistics for the data included mean ± standard deviation or median (interquartile range (IQR)) as appropriate, along with frequency and percentage values. The distribution of variables was assessed using the Kolmogorov–Smirnov and Shapiro–Wilk tests. The Independent Samples *t*-test was used to analyze normally distributed independent numerical data, while the Mann–Whitney U test was applied for non-normally distributed independent numerical data. The Chi-square test was used for categorical independent variables, and Fisher’s exact test was applied when Chi-square test assumptions were not met. Survival analysis was conducted using Cox regression (univariate and multivariate) and Kaplan–Meier methods. The effect size and cut-off value were determined using the ROC curve. Statistical analyses were performed using SPSS 28.0 software. A *p*-value of <0.05 was considered statistically significant.

## 3. Results

### 3.1. Characteristics of the Cohort

**Figure 1:**
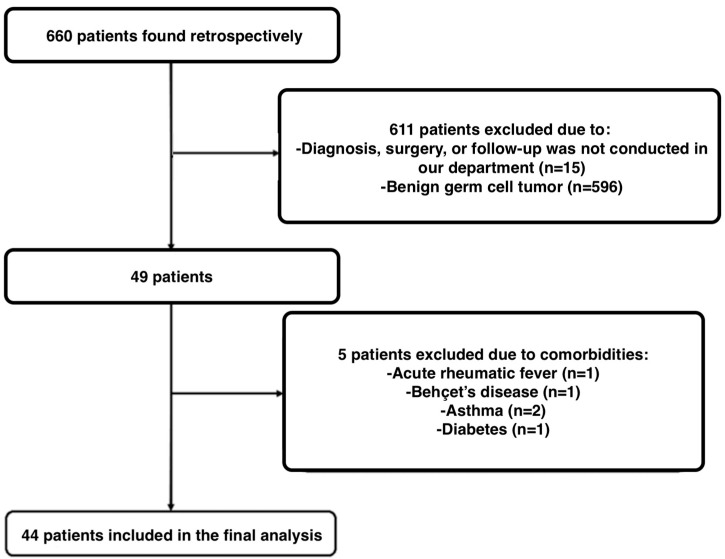
Flow-diagram of the study.

In our final analysis, we included 44 patients who met our inclusion and exclusion criteria (Figure 1). The mean age of the patients was 27 ± 13.5 years, with a mean weight of 63.0 ± 11.1 kg and a mean BMI of 23.5 ± 3.9 kg/m^2^. A total of 77.3% (34 patients) had a BMI below 25. One patient (2.3%) was pregnant. No comorbidities were identified in 84.1% (37 patients) of the study population.

The clinical and histopathological characteristics of our cohort are presented in Table 1. The most common type of germ cell tumor was dysgerminoma, found in 13 patients (29.5%), followed by immature teratoma in 9 patients (20.5%). The mean initial tumor size was 17.4 ± 7.3 cm. Most patients had lymphovascular space invasion, capsule invasion, necrosis, and ascites. Lymphadenectomy was performed in 29.5% of patients (13 patients) during surgery. Residual tumor was identified in 18.2% of patients (8 patients) following surgery, with a mean tumor size of 4.6 ± 3.6 cm. Most patients were at FIGO stage 1 (*n* = 21, 48%), stage 2 (*n* = 6, 14%), stage 3 (*n* = 15, 34%), and stage 4 (*n* = 2, 4.5%). Lymph node involvement was detected in 20.5% of patients (9 patients). Most patients received the BEP regimen as chemotherapy (54.5%). Only one patient was referred to nuclear medicine for radioactive iodine treatment for malignant struma ovarii. Five patients (11.4%) experienced chemotherapy-related side effects. During a mean follow-up period of 86.3 ± 76.4 months, recurrence was observed in 9 patients (20.5%), and 8 patients (18.2%) died (Table 1). In the overall cohort, the mean OS and DFS were 86.3 ± 76.4 months and 74.4 ± 77 months, respectively.

### 3.2. Determination of NLR Cut-Off Value for Predicting Survival During the Follow-Up Period

**Figure 2:**
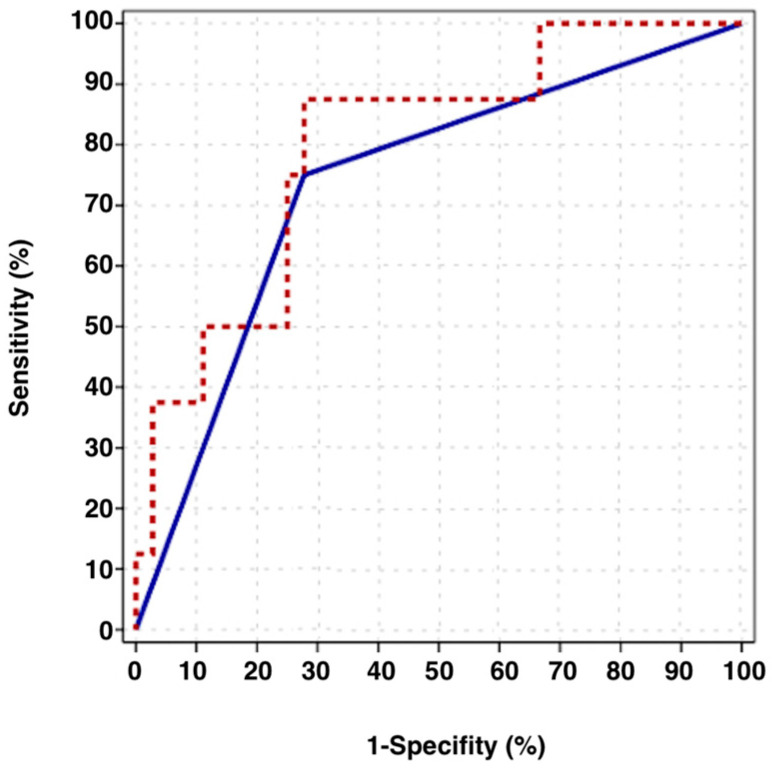
Neutrophil-to-lymphocyte ratio (NLR) cut-off value—receiver operating characteristic curve (ROC). The blue line represents the NLR cut-off value of 3.69, while the red dotted line represents the NLR.

NLR effectively distinguished between survived and deceased patients during the follow-up period, with an area under curve (AUC) of 0.799 (95% CI: 0.634–0.963, *p* = 0.009). A cut-off value of 3.69 was identified as an effective threshold, yielding an AUC of 0.736 (95% CI: 0.541–0.931, *p* = 0.039). At this cut-off, NLR showed a sensitivity of 75.0%, specificity of 72.2%, a positive predictive value of 37.5%, and a negative predictive value of 92.9% (Figure 2.)

### 3.3. Evaluation of Factors Affecting Overall Survival

Univariate analysis revealed that factors such as age, BMI, pregnancy, initial tumor size, residual tumor size, pathology, pathology subtype, lymphadenectomy, lymph node involvement, LVSI, capsule invasion, necrosis, ascites, recurrence, metastasis, chemotherapy, BEP regimen, and the number of chemotherapy cycles did not have a statistically significant impact on OS.

Univariate analysis revealed that weight, presence of comorbidity, residual tumor, FIGO stage, and chemotherapy side effects significantly impacted OS (*p* = 0.037, *p* = 0.019, *p* < 0.001, *p* = 0.029, and *p* = 0.035, respectively). Multivariate analysis indicated that presence of comorbidity and residual tumor significantly affected OS (*p* = 0.028 and *p* = 0.002, respectively) (Table 2).

Univariate analysis demonstrated that post-chemotherapy Hb and Htc values, preoperative neutrophil count, post-chemotherapy lymphocyte count, preoperative WBC count, post-chemotherapy beta-hCG, preoperative NLR, postoperative CEA, CA-125 levels both preoperatively and post-chemotherapy, as well as AFP levels at all three time points (preoperative, postoperative, and post-chemotherapy) had a statistically significant impact on OS (*p* = 0.014, *p* = 0.022, *p* = 0.001, *p* = 0.047, *p* = 0.003, *p* = 0.028, *p* = 0.001, *p* = 0.039, *p* = 0.028, *p* = 0.037, *p* = 0.010, *p* = 0.004, and *p* = 0.038, respectively) (Table 3). Multivariate analysis indicated that postoperative AFP values significantly affected OS (*p* = 0.004) (Table 3).

**Figure 3:**
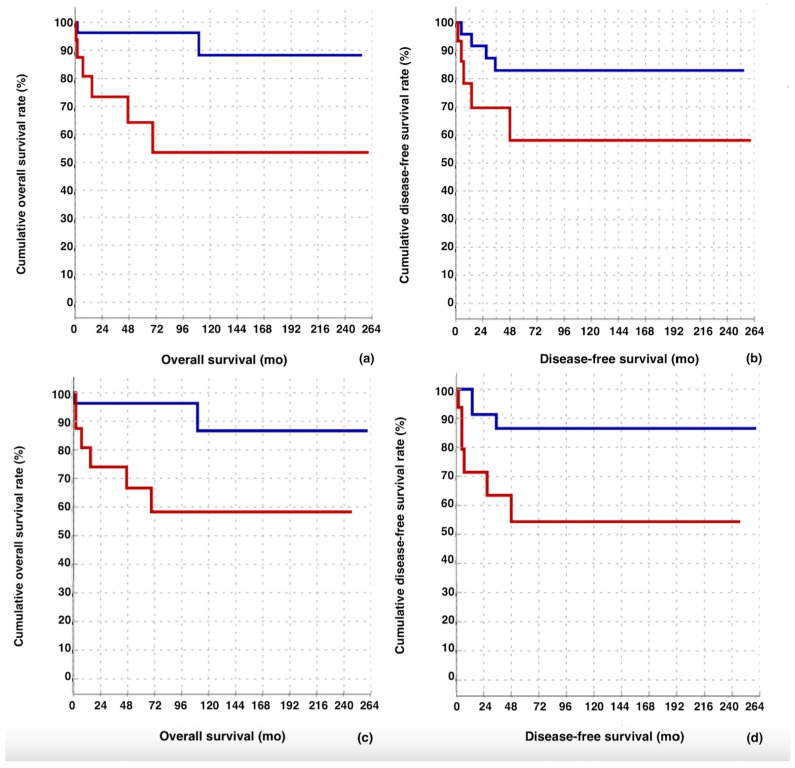
The effect of neutrophil-to-lymphocyte ratio (NLR) on (**a**) overall survival and (**b**) disease-free survival. The blue line represents NLR < 3.69, and the red line represents NLR ≥ 3.69. In addition, the effect of FIGO stage on (**c**) overall survival and (**d**) disease-free survival. The blue line represents early stage (FIGO I–II), and the red line represents late stage (FIGO stage III–IV).

The overall median survival in the group with NLR ≥ 3.69 (153.2 months) was found to be significantly lower than in the group with NLR < 3.69 (234 months) (*p* = 0.010) (Table 4) (Figure 3a). The median survival was significantly lower in the late stage group (155.0 months) compared to the early stage group (236.8 months) (*p* = 0.028). (Table 4) (Figure 3c).

### 3.4. Evaluation of Factors Affecting Disease-Free Survival

Univariate analysis showed no significant impact on DFS for weight, BMI, pregnancy, presence of comorbidity, initial tumor size, residual tumor, residual tumor size, pathology, pathology subtype, lymphadenectomy, lymph node involvement, LVSI, capsule invasion, necrosis, ascites, chemotherapy, chemotherapy regimen, BEP regimen, number of chemotherapy cycles, chemotherapy side effects, and metastasis (Table 2).

Univariate analysis revealed that age and FIGO stage significantly affected DFS (*p* = 0.010 and *p* = 0.029, respectively) (Table 2).

Univariate analysis showed that postoperative Hb and Htc, postchemotherapy Htc, post-chemotherapy beta-hCG, preoperative CEA, preoperative and post-chemotherapy CA-125, postchemotherapy CA-15-3, and postoperative AFP significantly affected DFS (*p* = 0.010, *p* = 0.015, *p* = 0.047, *p* = 0.017, *p* = 0.028, *p* = 0.029, *p* = 0.029, *p* = 0.042, and *p* = 0.009, respectively) (Table 3).

In the multivariate model, age and postoperative Hb values significantly affected survival duration (*p* = 0.025 and *p* = 0.025, respectively) (Table 2 and Table 3).

There was no significant difference in median DFS between the group with NLR ≥ 3.69 (159.3 months) and the group with NLR < 3.69 (215 months) (*p* = 0.094) (Table 4) (Figure 3b). The median DFS was significantly lower in the late-stage group (142.3 months) compared to the early stage group (228.7 months) (*p* = 0.023) (Table 4) (Figure 3d).

### 3.5. Comparison Between Variables Based on Neutrophil-to-Lymphocyte Ratio Cut-Off Value

There were no significant differences between the groups with NLR < 3.69 and NLR ≥ 3.69 regarding age, weight, BMI, pregnancy status, presence of comorbidity, initial tumor size, residual tumor size, FIGO stage, pathology, lymphadenectomy, LVSI, capsule invasion, necrosis, ascites, chemotherapy, BEP regimen, number of chemotherapy cycles, chemotherapy side effects, recurrence, metastasis, or follow-up period.

In the group with NLR ≥ 3.69, the presence of residual tumor was significantly higher compared to the NLR < 3.69 group (37.5% vs. 7.1%, *p* = 0.012). Lymph node involvement was also significantly more common in the NLR ≥ 3.69 group compared to the NLR < 3.69 group (37.5% vs. 10.7%, *p* = 0.034). Additionally, the mortality rate was significantly higher in the NLR ≥ 3.69 group (37.5% vs. 7.1%, *p* = 0.012).

### 3.6. The Difference in NLR Values over Time and in Comparison with Patients Who Died and Those with Recurrence

In the survivor group, postoperative NLR was significantly higher compared to preoperative values (*p* = 0.003), while post-chemotherapy NLR showed a significant decrease compared to preoperative levels (*p* = 0.001). The mean preoperative NLR values differed significantly between the survivor and deceased groups (*p* = 0.009) (Table 5). In the non-recurrent group, postoperative NLR increased significantly (*p* = 0.035), while post-chemotherapy NLR showed a significant decrease compared to both preoperative (*p* = 0.001) and postoperative (*p* = 0.002) values. In the recurrent group, post-chemotherapy NLR significantly decreased relative to preoperative values (*p* = 0.043) (Table 5).

## 4. Discussion

In our study, we confirmed the prognostic significance of NLR, highlighting its potential utility in individualized risk stratification and follow-up planning for patients with malignant ovarian germ cell tumors. We demonstrated that a higher preoperative NLR was significantly associated with reduced OS, but not with DFS. Moreover, elevated NLR levels were associated with a higher incidence of residual tumor, lymph node involvement, and increased mortality. These findings highlight the value of NLR—an easily accessible parameter obtained from routine complete blood counts—as a meaningful prognostic marker in survival analysis.

NLR has been studied as a prognostic marker in various cancers. For example, a study by Nemoto et al. on patients with metastatic colorectal cancer, found that OS was significantly better in those whose NLR decreased following chemotherapy [34]. Similarly, in our study, patients with malignant ovarian germ cell tumors who demonstrated longer survival showed a significant decrease in NLR values following chemotherapy. In contrast, a study on testicular germ cell tumors reported no significant difference in the prevalence of seminomas and non-seminomas between the NLR ≥ 4 and NLR < 4 groups [35]. Although standardized cut-off values have not yet been established, NLR continues to show promising potential as a simple and accessible prognostic marker.

The subtype of ovarian germ cell tumor and the presence of ascites are other prognostic factors for female malignant ovarian germ cell tumors. In a study by Sadigova et al., which evaluated the clinicopathological features and survival analysis of malignant ovarian germ cell tumors, mixed germ cell tumor pathology was identified as a poor prognostic factor [36]. Similarly, a study by Ayhan et al. reported that endodermal sinus tumor pathology was associated with poorer survival compared to other histological subtypes [37]. Guo et al. also identified histological type as a prognostic factor for survival [38]. In our study, the proportion of patients with dysgerminoma pathology was significantly lower in the deceased group than in the survivor group during the follow-up period; however, histological type was not found to be a significant factor in survival analysis. Although Yasui et al. reported that NLR values were higher in undifferentiated adenocarcinomas compared to differentiated carcinomas in early-stage gastric cancer [39], our study found no significant difference in pathology results between the NLR ≥ 3.69 and NLR < 3.69 groups. Furthermore, ascites is associated with the spread, recurrence, and treatment resistance of ovarian cancer [40]. In our study, the presence of ascites did not have a significant impact on survival. Additionally, no significant difference in ascites was observed between the NLR < 3.69 and NLR ≥ 3.69 groups. Therefore, NLR values cannot be recommended to distinguish between the subtypes of ovarian germ cell tumors or the presence of ascites.

Lymph node involvement has been evaluated as a prognostic factor in patients with malignant ovarian germ cell tumors. In a study by Kumar et al., lymph node metastasis was identified as a poor prognostic factor in these tumors [41]. However, in our study, lymph node involvement did not show any significant difference between deceased and survived patients, nor did it have a significant impact on OS or DFS. Similarly, a study by Mahdi et al. on early-stage malignant ovarian germ cell tumors also reported that lymph node metastasis was not associated with survival [42]. Although no significant difference was found in survival analysis, our study demonstrated that lymph node involvement was significantly higher in the NLR ≥ 3.69 group, suggesting that NLR might serve as an indirect marker of lymph node involvement.

Our multivariate analysis showed that postoperative residual tumor significantly affected OS, while age influenced DFS. Supporting our findings, a retrospective study by Won Lee et al., involving 57 patients, identified residual tumor as the only significant variable associated with treatment failure in multivariate analysis [43]. Conversely, in a study by Naioudis et al., macroscopic residual disease in advanced-stage malignant ovarian germ cell tumors was not associated with a poor prognosis [44]. We found that the rate of residual tumor was higher among patients who died during follow-up. Additionally, the presence of residual tumor was significantly more frequent in the NLR ≥ 3.69 group, reinforcing the potential utility of NLR as an indicator of residual tumor presence.

There is controversy regarding age and its prognostic significance in malignant ovarian germ cell tumors. In a study by Murugaesu et al. investigating prognostic factors and long-term outcomes in malignant ovarian germ cell tumors, age was not found to have prognostic significance [6]. Similarly, in a study by Salhi et al. on pure dysgerminomas, age above or below 15 years was reported to have no significant effect on OS or DFS [45]. Conversely, Guo et al. identified age as an independent prognostic factor for survival in malignant ovarian germ cell tumors [38]. Despite conflicting findings in the literature regarding age, our study showed that both univariate and multivariate analyses revealed a significant effect of age on DFS.

The limitations of our study include its retrospective nature and small sample size. However, this is the first study to determine the cut-off value of NLR in malignant ovarian germ cell tumors for survival prediction. NLR may vary due to factors such as patients’ existing comorbidities or inflammatory and infectious status at the time of sample collection. To minimize bias, we excluded patients with inflammatory conditions. However, since this is a retrospective study, preoperative blood count values may have been influenced by factors that were not always identifiable in the patients’ medical records. Although our exclusion criteria aimed to exclude patients with acute infections or inflammatory diseases based on the available medical information, we acknowledge that some confounding factors may not have been eliminated. NLR cut-off values have been reported at different levels across various studies [24,34]. This variability may be due to differences in cancer types, the timing of blood sample collection, patients’ comorbidities, the use of different treatment protocols, the presence of inflammation or infection at the time of sampling, smoking status, and the different methods used to calculate the cut-off values. In some studies, the NLR cut-off was determined using the ROC curve, while others used the median value, the log-rank test, interquartile ranges, or values derived from literature reviews. For instance, in a study on uterine sarcomas, the NLR threshold was determined to be 2.6 using the ROC curve, while in a study on testicular germ cell tumors, the threshold was found to be 4 using the median value [24,34]. In our study, the NLR threshold was determined to be 3.69 by the ROC curve.

## 5. Conclusions

Our study demonstrated that preoperative NLR significantly impacts OS in female patients with malignant ovarian germ cell tumors, with those in the NLR ≥ 3.69 group experiencing a markedly shorter survival time. However, no significant difference was observed in DFS, which may be due to the limited sample size. Given the retrospective design and small cohort of this study, further research with larger, prospective studies is necessary to validate these findings and better understand the prognostic value of NLR in this patient population.

## Figures and Tables

**Table 1:** Clinical and histopathological characteristics of malignant ovarian germ cell tumors of our cohort.

Variables			*n* (%) or Mean ± SD (*n* = 44)
Subtype of malign germ cell tumor	Dysgerminoma		13 (29.5%)
	Mixed Germ Cell Tumor		8 (18.2%)
	Yolk Sac		8 (18.2%)
	Immature Teratoma	Grade I	2 (4.5%)
		Grade II	5 (11.4%)
		Grade III	2 (4.5%)
	Embryonal Carcinomas		1 (2.3%)
	SCC Arising in Background of Teratoma		3 (6.8%)
	Malignant Struma Ovarii		2 (4.5%)
LVSI	(−)		14 (31.8%)
	(+)		30 (68.2%)
Presence of capsule invasion	(−)		13 (29.5%)
	(+)		31 (70.5%)
Initial tumor size (cm)			17.4 ± 7.3
Presence of necrosis	(−)		9 (20.5%)
	(+)		35 (79.5%)
Presence of ascites	(−)		14 (31.8%)
	(+)		30 (68.2%)
FIGO Stage	Stage 1	Stage 1A	9 (20.5%)
		Stage 1C	12 (27.3%)
	Stage 2	Stage 2A	1 (2.3%)
		Stage 2B	4 (9.1%)
		Stage 2C	1 (2.3%)
	Stage 3	Stage 3A	2 (4.5%)
		Stage 3B	2 (4.5%)
		Stage 3C	11 (25.0%)
	Stage 4		2 (4.5%)
Chemotherapy	(−)		18 (40.9%)
	(+)	BEP	24 (54.5%)
		Etoposide Cisplatin	1 (2.3%)
		Carboplatin Paclitaxel	1 (2.3%)
Number of chemotherapy cycles (*n* = 26)	II		2 (4.5%)
	III		13 (29.5%)
	IV		7 (15.9%)
	VI		4 (9.1%)
Chemotherapy side effects	(−)		39 (88.6%)
	(+)	Febrile Neutropenia	1 (2.3%)
		Pancytopenia + Febrile Neutropenia + Esophageal Candida	1 (2.3%)
		Paraneoplastic Dermatomyositis	1 (2.3%)
		Right Hemiplegia	1 (2.3%)
		Thrombocytopenia	1 (2.3%)
Metastasis	(−)		19 (43.2%)
	(+)		25 (56.8%)
Recurrence	(−)		35 (79.5%)
	(+)		9 (20.5%)
Death	(−)		36 (81.8%)
	(+)	Covid	1 (2.3%)
		Febrile Neutropenia	1 (2.3%)
		Sepsis	3 (6.8%)
		Sepsis + Pulmonary Embolism	1 (2.3%)
		Respiratory Failure	2 (4.5%)
Follow-up duration (mo)			86.3 ± 76.4

(−): absent; (+): present; BEP: Bleomycin, etoposide, and platinum; LVSI: lymphovascular space invasion; SD: standard deviation.

**Table 2:** Evaluation of the effect of clinical and histopathological variables on overall and Ddisease-free survival in univariate and multivariate model.

	Overall Survival			Disease-Free Survival		
	Univariate Model					
	HR	95% CI	*p*	HR	95% CI	*p*
Age (years)	1.038	0.092–1.087	0.109	1.075	1.017–1.136	**0.010**
Weight (cm)	1.048	1.003–1.095	**0.037**	1.013	0.958–1.071	0.656
BMI	1.109	0.999–1.231	0.052	1.017	0.875–1.181	0.830
Pregnancy	5.319	0.632–44.77	0.124	3.819	0.476–30.641	0.207
Presence of comorbidity	5.754	1.326–24.962	**0.019**	0.043	0.000–>100	0.563
Initial tumor size (cm)	1.075	0.983–1.175	0.113	1.005	0.913–1.107	0.919
Residual tumor	19.185	4.336–84.879	**<0.001**	5.073	0.999–25.765	0.050
Residual tumor size (cm)	0.956	0.755–1.209	0.704	0.674	0.326–1.392	0.287
FIGO Stage	2.340	1.092–5.012	**0.029**	2.110	1.081–4.120	**0.029**
Dysgerminoma-Non-dysgerminoma	44.633	0.109–>100	0.216	4.956	0.618–39.761	0.132
Pathology subtype	1.317	0.904–1.920	0.152	1.216	0.839–1.762	0.302
Lymphadenectomy	0.276	0.034–2.260	0.230	0.550	0.114–2.652	0.457
Lymph node involvement	1.117	0.223–5.599	0.893	0.485	0.060–3.886	0.495
LVSI	2.875	0.353–23.404	0.324	3.376	0.422–27.023	0.252
Capsule invasion	1.193	0.240–5.920	0.829	1.567	0.325–7.551	0.575
Presence of necrosis	1.202	0.146–9.934	0.864	0.692	0.143–3.342	0.647
Presence of ascites	34.474	0.050–>100	0.288	1.448	0.299–7.012	0.646
Chemotherapy	0.512	0.126–2.071	0.348	0.421	0.112–1.577	0.199
Chemotherapy regimen	3.654	0.372–35.856	0.266	3.322	0.343–32.137	0.300
BEP regimen	0.375	0.089–1.584	0.182	0.305	0.076–1.225	0.094
Number of chemotherapy cycles	0.907	0.641–1.284	0.583	0.793	0.557–1.128	0.196
Chemotherapy side effects	4.712	1.115–19.914	**0.035**	2.317	0.481–11.175	0.295
Recurrence	3.729	0.931–14.929	0.063	-	-	-
Metastasis	5.385	0.662–43.825	0.115	3.101	0.643–14.949	0.158
	**Overall Survival**			**Disease-free Survival**		
	**Multivariate Model**					
	**HR**	**95% CI**	***p***	**HR**	**95% CI**	***p***
Age	-	-	**-**	1.067	1.008–1.129	**0.025**
Presence of comorbidity	33.666	1.457–777.850	**0.028**	-	-	-
Residual tumor	51.847	4.196–640.676	**0.002**	-	-	-

All the variables were analyzed by Cox regression (forward LR). *p* < 0.05 was considered statistically significant (stated in bold text on the table). BEP: Bleomycin, etoposide, and platinum. BMI: Body-mass index. CI: Confidence interval. HR: Hazard ratio. LVSI: Lymphovascular space invasion.

**Table 3:** Evaluation of the effect of labarotary parameters on overall and disease-free survival in univariate and multivariate model.

		Mean ± SD	Overall Survival			Disease-Free Survival		
			Univariate Model					
			HR	% 95 CI	*p*	HR	% 95 CI	*p*
Hb (g/dL)	Postop	10.7 ± 1.2	0.981	0.553–1.741	0.948	0.484	0.278–0.841	**0.010**
	Post-CT	11.4 ± 1.5	0.405	0.197–0.833	**0.014**	0.539	0.291–1.000	0.050
Htc (%)	Postop	32.2 ± 3.2	0.944	0.773–1.154	0.575	0.813	0.688–0.960	**0.015**
	Post-CT	34.1 ± 4.1	0.753	0.590–0.960	**0.022**	0.799	0.640–0.997	**0.047**
Neutrophil (×10^3^/µL)	Preop	5609.5 ± 2677.1	1.000	1.000–1.000	**0.001**	1.000	1.000–1.000	0.888
Lymphocyte (×10^3^/µL)	Post-CT	1433.8 ± 432.3	0.997	0.995–1.000	**0.047**	0.998	0.995–1.000	0.098
WBC (×10^3^/µL)	Preop	7941.1 ± 3431.2	1.000	1.000–1.000	**0.003**	1.000	1.000–1.000	0.824
Beta-hCG (mIU/mL)	Post-CT	0.8 ± 1	2.028	1.078–3.817	**0.028**	1.911	1.123–3.250	**0.017**
NLR	Preop	3.5 ± 1.7	1.639	1.226–2.191	**0.001**	1.211	0.755–1.940	0.427
CEA (ug/L)	Preop	3.2 ± 4.8	1.095	0.982–1.221	0.102	1.188	1.019–1.385	**0.028**
	Postop	4.3 ± 10.7	1.036	1.002–1.071	**0.039**	1.090	0.994–1.196	0.068
Ca 125 (U/mL)	Preop	222.2 ± 297.1	1.001	1.000–1.003	**0.028**	1.002	1.000–1.003	**0.029**
	Post-CT	15.3 ± 10.2	1.125	1.007–1.256	**0.037**	1.101	1.010–1.200	**0.029**
Ca 15-3 (U/mL)	Post-CT	19.4 ± 9.8	1.077	0.993–1.169	0.074	1.104	1.003–1.215	**0.042**
AFP (ng/mL)	Preop	3966.9 ± 7002	1.000	1.000–1.000	**0.010**	1.000	1.000–1.000	0.562
	Postop	458.2 ± 895	1.001	1.000–1.001	**0.004**	1.001	1.000–1.001	**0.009**
	Post-CT	5.1 ± 8	1.064	1.004–1.128	**0.038**	1.054	0.997–1.114	0.063
			**Overall survival**			**Disease-free survival**		
			**Multivariate Model**					
			**HR**	**% 95 CI**	***p***	**HR**	**% 95 CI**	***p***
Hb (g/dL)	Preop		-	-	-	-	-	-
	Postop		-	-	-	0.517	0.290–0.922	**0.025**
	Post-CT		-	-	-	-	-	-
AFP (ng/mL)	Preop		-	-	-	-	-	-
	Postop		1.002	1.001–1.003	**0.004**	-	-	-
	Post-CT		-	-	-	-	-	-

The results for statistically non-significant parameters can be found in Supplemental Table S1. All the variables were analyzed by Cox regression (forward LR). *p* < 0.05 was considered statistically significant (stated in bold text on the table). AFP: Alpha-fetoprotein. Beta-hCG: Human chorionic gonadotropin. Ca 125: Cancer antigen 125. Ca 15-3: Cancer antigen 15-3. CEA: Carcinoembryonic antigen. CI: confidence interval. Hb: Hemoglobin. Htc: Hematocrit. HR: Hazard ratio. NLR: Neutrophil–lymphocyte ratio. Preop: Preoperative. Post-CT: Postchemotherapy. Postop: Postoperative. Plt: Platelet. RDW: Red cell distribution width. WBC: White blood cell.

**Table 4:** Comparison of median survival and median disease-free survival based on neutrophil-to-lymphocyte ratio cut-off value and FIGO stages.

		Median Survival (mo)	95% CI	*p*	Median Disease-Free Survival (mo)	95% CI	*p*
NLR	<3.69	234.0	206.2–261.8	**0.010**	215.0	179.2–250.8	0.094
	≥3.69	153.2	87.6–218.8		159.3	88.8–229.7	
Total		207.2	173.9–240.6		199.6	164.6–234.5	
FIGO Stage	Early (I–II)	236.8	204.7–269.0	**0.028**	228.7	194.6–262.8	**0.023**
	Late (III–IV)	155.0	97.8–212.2		142.3	79.2–205.3	
Total		207.2	173.9–240.6		199.6	164.6–234.5	

Kaplan–Meier (log rank). *p* < 0.05 was considered statistically significant (stated in bold text on the table). CI: Confidence interval; NLR: neutrophil-lymphocyte ratio; mo: months.

**Table 5:** Comparison of neutrophil-to-lymphocyte ratio (NLR) in relation to recurrence and mortality across different time points.

		Recurrence (−) (*n* = 35)	Recurrence (+) (*n* = 9)	*p*	Death (−) (*n* = 36)	Death (+) (*n* = 8)	*p*
		*n* (%) or Mean ± SD			*n* (%) or Mean ± SD		
NLR	Preop	3.49 ± 1.81	3.60 ± 1.33	0.590 *	3.13 ± 1.12	5.25 ± 2.73	0.009 *
	Postop	5.50 ± 4.50	7.55 ± 7.48	0.494 *	5.58 ± 4.42	7.44 ± 8.10	0.726 *
	Post-CT	2.05 ± 0.93	2.02 ± 1.15	0.770 *	2.03 ± 0.92	2.13 ± 1.17	0.649 *
**Change Over Time**							
Preop/Postop		2.01 ± 4.84	3.94 ± 8.04	0.673 *	2.45 ± 4.75	2.20 ± 8.88	0.315 *
Intragroup change *p*		0.035 **	0.314 **		0.003 **	0.779 **	
Preop/Post-CT		−1.34 ± 1.12	−2.17 ± 1.71	0.380 *	−1.32 ± 1.12	−2.24 ± 1.66	0.269 *
Intragroup change *p*		0.001 **	0.043 **		0.001 **	0.043 **	
Postop/Post-CT		−2.61 ± 3.89	−4.82 ± 8.26	0.974 *	−2.83 ± 3.79	−3.93 ± 8.70	0.313 *
Intragroup change *p*		0.002 **	0.225 **		0.471 **	0.686 **	

* Mann–Whitney u test. ** Wilcoxon test. *p* < 0.05 was considered statistically significant (stated in bold text on the table). NLR: Neutrophil–lymphocyte ratio. Preop: Preoperative. Post-CT: Postchemotherapy. Postop: Postoperative. SD: Standard deviation.

## Data Availability

The raw data supporting the conclusions of this article will be made available by the authors on request.

## Supplementary Materials

The following supporting information can be downloaded at: https://www.mdpi.com/article/10.3390/diagnostics15081040/s1, Table S1. Evaluation of the effect of laboratory parameters variables on overall and disease-free survival in univariate and multivariate model.
