# Supplementary material for: The Effect of Neutrophil-to-Lymphocyte Ratio on Prognosis in Malignant Ovarian Germ Cell Tumors
Source: Diagnostics (Basel). 2025 Apr 19;15(8):1040. doi: 10.3390/diagnostics15081040 (PMC12025752; doi:10.3390/diagnostics15081040)
Supplement: Supplementary file 1 [file diagnostics-15-01040-s001.zip › diagnostics-3571102-supplementary.pdf]

Supplemental Table S1. Evaluation of the Effect of Laboratory Parameters Variables on Overall and Disease-Free Survival in Univariate and Multivariate Model

		Mean±SD	Median (IQR)	Overall survival			Disease-free survival		
				Univariate Model					
				HR	% 95 CI	p	HR	% 95 CI	p
Hb (g/dL)	Preop	11.5±1.3	11.5 (11-12)	0.825	0.476-1.427	0.491	1.096	0.669-1.797	0.715
	Postop	10.7±1.2	10.7 (9.9-11.5)	0.981	0.553-1.741	0.948	0.484	0.278-0.841	**0.010**
	Post-CT	11.4±1.5	11.4 (10.3-12.6)	0.405	0.197-0.833	**0.014**	0.539	0.291-1.000	0.050
Htc (%)	Preop	35.1±3.6	35.1 (33-37.2)	0.952	0.786-1.153	0.614	1.108	0.923-1.328	0.271
	Postop	32.2±3.2	32 (29.9-33.8)	0.944	0.773-1.154	0.575	0.813	0.688-0.960	**0.015**
	Post-CT	34.1±4.1	34.1 (30.9-37)	0.753	0.590-0.960	**0.022**	0.799	0.640-0.997	**0.047**
Plt (x10³/μL)	Preop	375.3±95.2	370 (309.5-412)	1.000	1.000-1.000	0.590	1.000	1.000-1.000	0.844
	Postop	322.3±121.5	315.5 (235.2-381.5)	1.000	1.000-1.000	0.297	1.000	1.000-1.000	0.218
	Post-CT	300±107.1	281 (230-320)	1.000	1.000-1.000	0.271	1.000	1.000-1.000	0.541
RDW (%)	Preop	15.6±2.4	15.3 (13.7-17.2)	1.042	0.771-1.407	0.790	0.752	0.519-1.090	0.132
	Postop	17.5±3.5	17.1 (14.4-19.8)	1.117	0.922-1.354	0.257	1.122	0.942-1.336	0.199
	Post-CT	17.8±2.5	17.6 (15.9-19.6)	1.104	0.772-1.580	0.587	1.124	0.810-1.561	0.485
Neutrophil (X10³/μL)	Preop	5609.5±2677.1	5500 (3925-6000)	1.000	1.000-1.000	**0.001**	1.000	1.000-1.000	0.888
	Postop	6757.5±3996.5	5830 (3895-8415)	1.000	1.000-1.000	0.166	1.000	1.000-1.000	0.345
	Post-CT	3088.1±1469.1	2980 (2380-3537.5)	1.000	0.999-1.001	0.826	1.000	0.999-1.000	0.733
Lymphocyte (X10³/μL)	Preop	1774.5±981	1700 (1240-2067.5)	1.000	1.000-1.001	0.220	1.000	0.998-1.001	0.540
	Postop	1466.2±659.7	1415 (1081.3-1800)	1.000	0.999-1.001	0.674	0.999	0.998-1.001	0.396
	Post-CT	1433.8±432.3	1475 (1142.5-1700)	0.997	0.995-1.000	**0.047**	0.998	0.995-1.000	0.098
WBC (X10³/μL)	Preop	7941.1±3431.2	7200 (6000-8642.5)	1.000	1.000-1.000	**0.003**	1.000	1.000-1.000	0.824
	Postop	8733.6±4188.1	7550 (6122.5-10637.5)	1.000	1.000-1.000	0.071	1.000	1.000-1.000	0.426
	Post-CT	4825.8±1892.1	4640 (3722.5-5462.5)	1.000	0.999-1.000	0.132	1.000	0.999-1.000	0.118
LDH (U/L)	Preop	747.1±624.5	576 (269-1000)	1.000	0.999-1.001	0.746	1.000	0.998-1.001	0.485
	Postop	490±449.8	300 (201.3-648)	1.000	0.999-1.002	0.573	0.999	0.996-1.001	0.279
	Post-CT	289.5±161.3	231 (162.5-359)	0.999	0.993-1.005	0.735	1.000	0.995-1.005	0.943
Beta-hCG	Preop	70.4±150.5	1.7 (0.1-99.2)	0.959	0.892-1.032	0.266	0.993	0.980-1.005	0.249
	Postop	1.4±1.5	1.2 (0.1-2.6)	1.369	0.968-1.936	0.076	1.282	0.903-1.822	0.165

(mIU/mL)	Post-CT	0.8±1	0.6 (0.1-1.2)	2.028	1.078-3.817	**0.028**	1.911	1.123-3.250	**0.017**
NLR	Preop	3.5±1.7	3.3 (2.2-4)	1.639	1.226-2.191	**0.001**	1.211	0.755-1.940	0.427
	Postop	5.9±5.2	4.1 (2.5-7.1)	1.084	0.969-1.214	0.160	1.109	0.994-1.238	0.063
	Post-CT	2±0.9	2 (1.7-2.2)	1.284	0.491-3.357	0.610	1.025	0.408-2.575	0.957
CEA (*ug/L*)	Preop	3.2±4.8	1.7 (0.8-4.1)	1.095	0.982-1.221	0.102	1.188	1.019-1.385	**0.028**
	Postop	4.3±10.7	1.9 (1.2-4.3)	1.036	1.002-1.071	**0.039**	1.090	0.994-1.196	0.068
	Post-CT	2.4±3	1.9 (1.1-2.5)	1.341	0.994-1.810	0.055	1.357	0.995-1.852	0.054
Ca 125 (U/mL)	Preop	222.2±297.1	164.6 (51.6-320)	1.001	1.000-1.003	**0.028**	1.002	1.000-1.003	**0.029**
	Postop	69.6±74.4	53.2 (13.5-94.8)	1.006	1.000-1.014	0.069	0.999	0.990-1.009	0.872
	Post-CT	15.3±10.2	15 (9.9-18.9)	1.125	1.007-1.256	**0.037**	1.101	1.010-1.200	**0.029**
Ca 19-9 (U/mL)	Preop	101.4±299.5	29.9 (10.5-99.5)	0.986	0.968-1.004	0.121	0.997	0.987-1.008	0.606
	Postop	11.5±10.4	9.9 (5.6-13.1)	0.924	0.821-1.041	0.193	0.961	0.873-1.059	0.426
	Post-Ct	10.4±8.1	9.8 (4.8-11.3)	0.901	0.747-1.087	0.278	0.876	0.717-1.071	0.197
Ca 15-3 (U/mL)	Preop	75.1±231.5	26.5 (16.2-71.7)	0.997	0.982-1.013	0.726	0.996	0.980-1.013	0.650
	Postop	29.2±35.4	19.8 (12.8-29.8)	1.009	0.993-1.024	0.281	1.008	0.992-1.024	0.329
	Post-CT	19.4±9.8	18.9 (16-20.5)	1.077	0.993-1.169	0.074	1.104	1.003-1.215	**0.042**
AFP (ng/mL)	Preop	3966.9±7002	1040.5 (2.1-4115)	1.000	1.000-1.000	**0.010**	1.000	1.000-1.000	0.562
	Postop	458.2±895	5.7 (2.3-580)	1.001	1.000-1.001	**0.004**	1.001	1.000-1.001	**0.009**
	Post-CT	5.1±8	3.7 (1.8-5)	1.064	1.004-1.128	**0.038**	1.054	0.997-1.114	0.063

		**Overall survival**			**Disease-free survival**		
		**Multivariate Model**					
		**HR**	**% 95 CI**	**p**	**HR**	**% 95 CI**	**p**
Hb (g/dL)	Preop	-	-	-	-	-	-
	Postop	-	-	-	0.517	0.290-0.922	**0.025**
	Post-CT	-	-	-	-	-	-
AFP (ng/mL)	Preop	-	-	-	-	-	-
	Postop	1.002	1.001-1.003	**0.004**	-	-	-
	Post-CT	-	-	-	-	-	-

All the variables were analyzed by Cox Regression (Forward LR).
$p < 0.05$ was considered statistically significant (stated in bold text on the table).

AFP: Alpha-fetoprotein, Beta-hCG: Human chorionic gonadotropin, Ca 125: Cancer antigen 125, Ca 15-3: Cancer Antigen 15-3, Ca 19-9: Cancer Antigen 19-9, CEA: Carcinoembryonic antigen, CI: confidence interval, Hb: Hemoglobin, Htc: Hematocrit, HR: Hazard ratio, LDH: Lactate Dehydrogenase, NLR: Neutrophil-Lymphocyte Ratio, Preop: Preoperative, Post-CT: Postchemotherapy, Postop: Postoperative, Plt: Platelet, RDW: Red cell distribution width, WBC: White Blood Cell.
